# The Role of Nutrients in Prevention, Treatment and Post-Coronavirus Disease-2019 (COVID-19)

**DOI:** 10.3390/nu14051000

**Published:** 2022-02-26

**Authors:** Maria Letizia Motti, Domenico Tafuri, Lorenzo Donini, Maria Teresa Masucci, Valentina De Falco, Filomena Mazzeo

**Affiliations:** 1 Department of Movement Sciences and Wellbeing, University “Parthenope”, 80133 Naples, Italy; domenico.tafuri@uniparthenope.it (D.T.); lorenzo.donini@uniparthenope.it (L.D.); filomena.mazzeo@uniparthenope.it (F.M.); 2 Neoplastic Progression Unit, Istituto Nazionale Tumori IRCCS “Fondazione G. Pascale”, 80131 Naples, Italy; m.masucci@istitutotumori.na.it; 3 Istituto di Endocrinologia e Oncologia Sperimentale del CNR, Dipartimento di Medicina Molecolare e Biotecnologie mediche, Università degli Studi di Napoli Federico II, Via Pansini 5, 80131 Napoli, Italy; valentina.defalco@ieos.cnr.it

**Keywords:** pandemic, COVID-19, nutritional supplement, SARS-CoV-2, Long-COVID, ARDS, inflammation, immune system

## Abstract

SARS-CoV-2 virus, infecting human cells via its spike protein, causes Coronavirus disease 2019 (COVID-19). COVID-19 is characterized by shortness of breath, fever, and pneumonia and is sometimes fatal. Unfortunately, to date, there is still no definite therapy to treat COVID-19. Therefore, the World Health Organization (WHO) approved only supportive care. During the COVID-19 pandemic, the need to maintain a correct intake of nutrients to support very weakened patients in overcoming disease arose. The literature available on nutrient intake for COVID-19 is mainly focused on prevention. However, the safe intake of micro- and/or macro-nutrients can be useful either for preventing infection and supporting the immune response during COVID-19, as well as in the post-acute phase, i.e., “long COVID”, that is sometimes characterized by the onset of various long lasting and disabling symptoms. The aim of this review is to focus on the role of nutrient intake during all the different phases of the disease, including prevention, the acute phase, and finally long COVID.

## 1. Introduction

The etiological agent of COVID-19 (Coronavirus Disease 19) was named SARS-CoV-2 (Severe Acute Respiratory Syndrome Coronavirus 2) on 11 February 2020, by the World Health Organization (WHO) [1].

SARS-CoV-2 was detected for the first time in December of 2019 in in Wuhan (Hubei), China [2]. From there, SARS-CoV-2 spread throughout the world by rapid viral person-to-person infection [3].

The virus reached nearly all countries in the world in less than six months and on 30 January 2020, it was named “the first pandemic of the 21st century” by the World Health Organization (WHO) [4].

SARS-CoV-2 is an envelope RNA- virus with a positive sense RNA genome (30 kb long) encoding four structural proteins, spike (S), envelope (E), membrane (M), and nucleocapsid (N). It has in total 11 genes, with 11 open reading frames (ORFs) [5].

The SARS-CoV-2 enters and infects nasal ciliated and lung alveolar epithelial cells, small intestine enterocytes, arterial and venous endothelial cells, and arterial smooth muscle cells via the interaction of its S protein with angiotensin converting enzyme 2 (ACE2) receptor [6].

Spike is a homotrimeric glycoprotein constituted by two functional subunits: S1 subunit that binds to ACE2 receptor and S2 subunit implicated in the fusion of the viral and host cell membranes. After the binding of the S1 subunit to ACE2 on human cells, the transmembrane protease serine 2 (TMPRSS2), in human cell membranes, cleaves the spike protein, activating the S2 domain. This process causes the virus to fuse with the cell and enter it [7,8].

**Figure 1:**
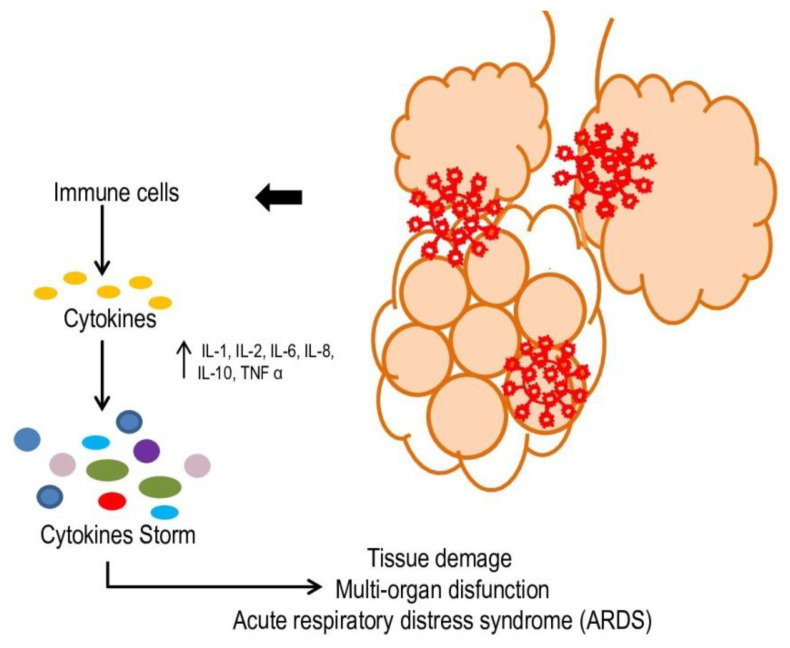
SARS-CoV-2 pathogenesis. In more aggressive cases of COVID-19, the virus can enter the bloodstream inducing an imbalance in the immune system, causing a cytokine storm. This can lead to tissue damage, ARDS and multi-organ dysfunction.

The infection by SARS-CoV2 of epithelial cells of the upper respiratory tract results in a local immune response caused by interferon beta (IFN-β) and CXC motif chemokine ligand 10 (CXCL-1O), giving mild symptoms, such as cough, fever, and rhinorrhea [9]. However, when SARS-CoV-2 enters the pulmonary alveoli, severe pneumonia can develop: the virus can infect type II alveolar cells, destroying them and causing widespread damage to the alveoli [10]. In very aggressive cases of COVID-19, the virus can enter the bloodstream and infect endothelial and other target cells in the kidneys, esophagus, bladder, ileum, heart tissues, and central nervous system. In patients in critical condition, the release of a large number of interleukins and cytokines, such as IL-1, IL-2, IL-6, IL-8, IL-10, CCL3, IP-10, and TNF α, causes the so-called “cytokine storm” (Figure 1) [11,12,13], which attracts neutrophils, CD4 helper, and CD8 cytotoxic lymphocytes, which, acting against the infection, generate a constant inflammatory state. This inflammatory situation causes apoptosis and necrosis of the surrounding tissue and self-feeds, causing damage to type I and type II alveolar cells. This promotes an increase in blood vessel permeability, leading to acute respiratory distress syndrome (ARDS) (Figure 1) [13,14,15].

In the last year, the development of vaccines has given great hope for countering the pandemic. Unfortunately, it has been shown that, in most cases, they do not fully protect against infection and spread of the virus. However, vaccine development has been the most important approach to preventing severe COVID-19 and plays a pivotal role in the control and reduction of mortality [16]. The virus uses its spike protein to enter the host cells by interacting with a specific receptor ACE2. Thus, all vaccines available so far target the spike protein. Vaccines induce the expression of the spike protein in the human cells, via mRNA or an adenoviral vector. The human immune system will recognize this protein as foreign and induce the production of neutralizing antibodies [16].

Recent studies proposed therapies for COVID-19 patients based on the pathogenesis of the infection and dependent on the course of the disease. In fact, they propose the use of oxygen therapy to counteract low oxygen saturation, anti-inflammatories to prevent the storm of cytokines and ARDS, antiviral agents to block as much as possible the entry of the virus into host cells, antithrombotic drugs to prevent cases of intravascular coagulation disseminated, and finally the use of monoclonal antibodies in the most serious patients [17,18]. Anti-SARS-CoV-2 monoclonal antibodies target the viral spike protein, preventing the virus from binding to host cells, and represent one of the primary immune responses against SARS-CoV-2 [18].

However, SARS-CoV-2 exerts its adaptive capacities through mutations of its protein, which gives the virus a competitive advantage, affecting its pathogenic potential. In fact, only a single variation of amino acid is able to influence the viral replication, transmission, or immune control evasion, making the vaccine ineffective. SARS-CoV-2, like other RNA viruses that typically have higher mutation rates than DNA viruses, selects genetic mutations, allowing a greater transmissibility in human cells and developing different variants. So, variants of the virus continue to be selected, presenting a variable number of mutations in the spike protein. The latest variant identified, Omicron, contains more than thirty amino acid mutations in the spike protein, mostly present in the receptor binding domain, and exhibits an increased transmissibility and escape ability from vaccines and therapies [19,20].

The progressive selection of new mutations will compromise the efficacy of both vaccines and monoclonal antibodies.

Given the rapid transmission, the severity of infections, and the absence of drugs able to completely inhibit SARS-CoV-2 infection, it is urgent to discover and develop molecules capable of improving the host’s natural defenses and further antiviral agents. The innovation of this review is represented by the focus on the three different moments of the infection, in particular long COVID, which has become increasingly worrying. In fact, in the initial phase of the pandemic, not enough time had elapsed to highlight these long-lasting symptoms, following the acute infection, which were sometimes confused with other diseases. Moreover, last but not least, the use of micro and macronutrients is a relatively inexpensive and easily manageable treatment, without hospitalization. If proven effective, it has the potential to change the course of the COVID-19 pandemic.

## 2. Materials and Methods

An online survey on PubMed and Scopus from beginning of COVID-19 pandemic to January 2022 of all scientific publications (case reports, letters to editors, reviews, original research) focused on SARS-CoV-2 was carried out. Search keywords were: COVID-19, nutrients, dietary supplements, SARS-CoV-2, long COVID, ARDS, inflammation, immune system. The online survey was carried out by a systematic analysis and a critical evaluation of the collected studies, considering the most valuable studies in the best journals. Only articles reporting associations between nutrients and COVID-19 in humans were analysed. All articles not published in English, articles with a short commentary, short notes, or incomplete results were excluded. Search results were screened for inclusion and exclusion criteria by all the authors. As the topic is very innovative, all the works found are very recent and some studies could remain incomplete.

## 3. Results

### 3.1. Nutrients in COVID-19 Prevention

During the COVID-19 pandemic, people trusted web and social media guidelines on the use of natural substances and supplements, miraculously acting in the prevention and treatment of COVID-19.

Some of these substances are already known as immune system enhancers. Many vitamins, including vitamins A, B6, B12, C, D, E, and folates as well as trace elements, including zinc, iron, selenium, magnesium, and copper, are very useful in supporting both innate and adaptive immune immunity. Deficiencies in these substances negatively affect the activation of the immune system in infections [21,22,23].

For example, vitamin C has a role in immune response, regulating several cellular functions of innate and adaptive immunity. Vitamin C influences neutrophil chemotaxis, phagocytosis, and the generation of reactive oxygen species. Furthermore, vitamin C promotes the differentiation and proliferation of B and T cells. It is known that vitamin C deficiency negatively regulates the immune response, making the body more sensitive to infections [21].

Vitamin D plays a role in the defense against viral infections as it is able to modulate both the adaptive and the innate immune systems [24,25,26]. It protects against respiratory pathogens by various mechanisms. Moreover, 25-hydroxyvitamin, the main circulating metabolite of vitamin D, responds to both viral and bacterial stimuli by inducing the expression of antimicrobial peptides [27,28,29]. In fact, various studies describe a correlation between low serum concentrations of 25-hydroxyvitamin D and an increased chance of contracting acute infections of the respiratory tract [30,31]. Moreover, the vitamin D metabolites induce autophagy and the synthesis of reactive intermediates of both nitrogen and oxygen, reducing acute respiratory infections and pneumonia [32,33,34].

Furthermore, vitamin B6 activates innate and adaptive immunity by influencing the proliferation of immune cells [35], Zn influences the development and activity of both neutrophils and NK cells [36], Fe regulates the differentiation and proliferation of T lymphocytes and, through the production of reactive oxygen species, plays a role in the removal of infectious agents [37]. Finally, selenium supports the activity of the immune system, as the deficiency of Se compromises both innate and acquired immunity [38]. Other nutrients such as omega-3 fatty acids (N-3 PUFAs) also participate in the effectiveness of the immune response, playing a role in reducing inflammation: they inhibit leukocyte chemotaxis, production of inflammatory cytokines, and T lymphocyte reactivity. Moreover, they give rise to resolvins and protectins that participate in the resolution of inflammation [39].

Most of these micronutrients are included in the European Union Register on Nutrition and Health Claims, as they play a role in the functioning of the immune system [40].

Therefore, various authors recommend the early use of substances, such as zinc, selenium, and vitamin D [41], as well as other micronutrients, to increase resistance to COVID-19 [42]. This should be done especially in areas at highest risk of developing COVID-19 and as early as possible in the case of suspected infections [43].

**Figure 2:**
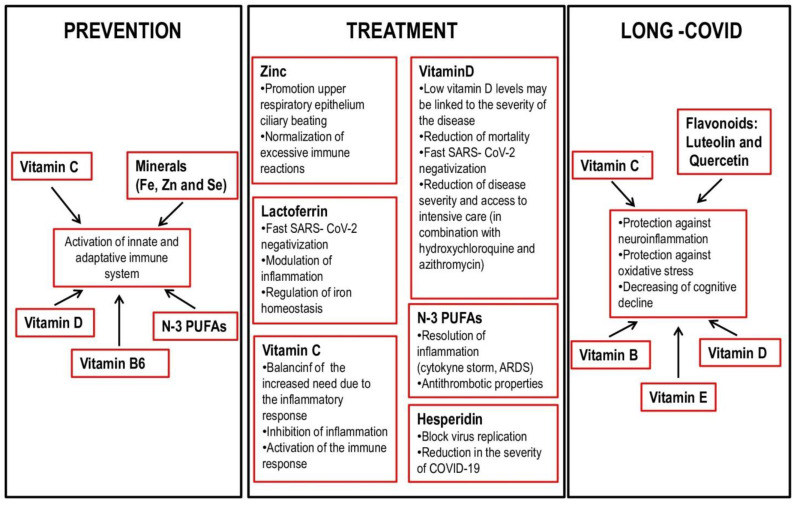
Functions of nutrients in prevention, treatment and post-COVID 19.

The World Health Organization reiterated during the pandemic the indications for proper nutrition based on the guidelines already known, which recommend a Mediterranean diet, with a prevalent consumption of fresh and unprocessed foods, vegetables, and in which the use of sugars and saturated fats and an excessive amount of salt are not recommended [44]. In the literature, there are several studies that highlight the important role of nutrition in the correct functioning of the immune response (Figure 2). Sometimes a correct diet can be sufficient to guarantee the correct intake of the micro and macro nutrients suggested in the study. However, for some of them, such as vitamin D, diet alone cannot increase serum 25 (OH) D concentrations to provide optimal protection from SARS-CoV-2. In addition, further randomized trials with an early administration of high doses of vitamin D after the onset of COVID-19 should be conducted in order to identify the right time to start vitamin D administration to achieve a significant effectiveness [45].

### 3.2. Nutrients in COVID-19 Treatment

Since SARS-CoV-2 infection became a pandemic, affecting millions of people, an urgent need has arisen for effective treatments against this disease.

COVID-19 patients, especially hospitalized ones, show strong consequences, such as hypermetabolism and muscle catabolism, due to a marked systemic inflammation, with a reduction in food intake and therefore malnutrition. Some studies show that the outcome of COVID-19 patients is correlated with their nutritional status [46,47].

Some data have shown that the lack of some minerals and vitamins has a negative effect on the patient’s recovery during the treatment of COVID-19 [48]. In fact, some micronutrients influence the production of inflammatory mediators during the disease and act as immunostimulants, so they are recommended for COVID-19 patients [48,49].

For this reason, many studies have focused on the role of micronutrients in supporting the treatment of COVID [41,48,50] (Figure 2).

#### 3.2.1. Vitamin C

During infections, vitamin C levels decrease because metabolism requires a great amount of this vitamin due to increasing inflammation. Vitamin C supplementation is used in the prevention and therapy of respiratory and systemic infections. For prevention, plasma levels of vitamin C of at least 100–200 mg/day are required. However, for the treatment of infection, higher doses of vitamin C are needed to balance the increased demand due to the inflammatory response [51].

Vitamin C in COVID-19 pathology inhibits inflammation and activates the immune response by acting on various mechanisms: it regulates the production of cytokines, the amount of released histamines, mitigates oxidative stress, and acts on the differentiation and proliferation of T and B lymphocytes [51,52].

However, the data on the effect of Vitamin C administration in the treatment of COVID-19 need further and larger prospective randomized studies [53].

#### 3.2.2. Vitamin D

The importance of vitamin D in COVID 19 prophylaxis has been previously described, and its role in modulating both the adaptive and innate immune systems has been highlighted [25,26].

The idea that low vitamin D levels may be linked to the severity of the disease has stimulated many studies. In a study carried out in India, severe COVID-19 patients presented with vitamin D deficiency with lower serum levels of 25-hydroxyvitamin D and with higher levels of inflammatory markers compared to asymptomatic patients [54].

In addition, other observational studies show that low levels of 25-hydroxyvitamin are associated with the severity of COVID-19 [55,56,57,58].

There are several data, albeit not definitive, that attribute a therapeutic function to vitamin D in COVID-19. In a first study, patients treated with a high-dose cholecalciferol supplementation showed greater SARS- CoV-2 negativization than those who do not have supplementation. [59]. Another one, highlights that the early use of calcifediol or cholecalciferol correlates with increased survival among COVID-19 hospitalized patients [60]. 

Other groups showed that a good vitamin D status reduces the use of intensive care and leads to a reduction in mortality [61,62]. In particular, some of these showed an association between the treatment of hospitalized COVID-19 patients with cholecalciferol and reduced mortality, regardless of initial 25-hydroxyvitamin D levels [63,64]. 

Another Spanish study has shown that early administration of high-dose calcifediol in combination with hydroxychloroquine and azithromycin greatly reduces the disease severity and access to intensive care compared to treatment with hydroxychloroquine or azithromycin alone [65].

However, although a sure correlation between vitamin D and recovery from COVID-19 has not yet been demonstrated, new guidelines have been provided in many countries recommending vitamin D supplementation in case of SARS-CoV-2 positivity.

#### 3.2.3. Zinc

Upper respiratory system virus infections are prevented by the presence of mucus and vibrating cilia [66]. SARS-CoV-2 infection causes damage to the ciliated epithelium and ciliary dyskinesia [67]. Zinc is essential in keeping tissue barriers intact and functioning. In fact, it is known that zinc promotes ciliary beating and, in rats with a Zn deficiency, its integration caused an improvement in both the number and length of the bronchial vibratory cilia [68,69]. Zinc is known to act on viral replication in several types of viruses, including Coronaviridiae [70]. It prevents fusion with the host cell membranes, inhibits viral polymerase activity, interferes with viral protein synthesis, inhibits the release of viral particles, and makes unstable the viral envelope [71]. COVID-19 is characterized by an imbalance of the immune response [72]. The most severe forms are characterized by systemic inflammation, due to cytokine storm, and organ failure. Moreover, some patients develop acute respiratory distress syndrome (ARDS) [73]. Zinc plays an important role in the immune response by normalizing excessive immune reactions, balancing the interactions among the various cell types of the immune system. Hence, zinc at high concentrations of inflammatory mediators prevents the destruction of host tissue [11].

However, further studies are needed to show that zinc can be used as a therapy for COVID-19. Despite several studies that have focused on using zinc supplementation alone or in combination with other drugs including hydroxychloroquine, results are not yet known and the effectiveness of zinc is still uncertain [74].

#### 3.2.4. N-3 PUFAs

It has been shown that N-3 PUFAs, commonly called omega-3 fatty acids, have excellent effects in fighting viral infections [74] and that their deficiency can cause a delayed resolution of inflammations [75,76].

Indeed, N-3 PUFAs play a valuable role in the therapy of inflammation associated diseases. Physiologically, the inflammation resolves quickly in the final phase of the immune response when negative feedback mechanisms are activated. Among these processes is the enzymatic conversion of omega-3 eicosapentaenoic acid (EPA) and docosahexaenoic acid (DHA) into specialized pro-resolution mediators (SPM) known as resolvins, protectins, and maresins, at the site of inflammation. SPMs participate, together with other molecules, in the resolution of inflammation, also in the respiratory tract [77,78]. Therefore, a plausible hypothesis could be that SPMs can help solve the cytokine storm and COVID-19 associated lung inflammation [79,80]. Before the COVID pandemic, N-3 PUFAs had been attributed a role in countering ARDS and sepsis [81,82,83] and since ARDS and sepsis characterize severe COVID patients [83], it is possible that N-3 PUFAs could represent a valid treatment in severe patients. This could be very important in the context of severe COVID-19 manifesting an uncontrolled inflammation, the so-called cytokine storm and ARDS [84].

Another mechanism by which N-3 PUFAs reduce inflammation levels is inhibiting leukocyte chemotaxis, the expression of adhesion molecules, and interaction between leucocytes and endothelium [39,85,86]. Moreover, N-3 PUFAs affect the adaptive immune response [85] by regulating antigen presentation and CD4^+^ Th1 cell production [87].

However, preliminary data on the correlation between omega-3 fatty acid intake and recovery from COVID-19 are still controversial. In a pilot study, blood omega-3 concentration from 100 COVID-19 patients was inversely related to the risk of death [88]. Therefore, although there are strong indications that N-3 PUFAs may play a role in regulating the immune system in COVID-19, existing data from randomized controlled trials (RCTs) are not always significant, so further studies are needed.

Finally, another positive effect of N-3 PUFAs in COVID-19 patients could be ascribed to their antithrombotic properties [89,90,91]. As some COVID-19 patients, particularly those with comorbidities, may develop complications, such as arterial and venous thrombosis [92], N-3 fatty acids may play an important role in the therapy of thrombotic complications from COVID-19.

#### 3.2.5. Lactoferrin

Lactoferrin, a milk-derived 80-kDa glycoprotein [93,94], synthesized by neutrophils, is involved in innate immunity and plays a role in host defense [95,96,97]. It is able to bind free iron [98] and is capable of modulating the tissue inflammatory process by inducing a decrease in the production of proinflammatory cytokines and by regulating the expression of some proteins involved in inflammatory and iron homeostasis (including ferroportin, membrane-bound ceruloplasmin, cytosolic ferritin, transferrin receptor 1) [99].

In COVID-19 patients, the release of proinflammatory cytokines such as interleukin (IL) -6 could both induce coagulopathy and affect iron homeostasis [100,101,102,103]. So Lactoferrin (Lf) could counteract SARS-CoV-2 infection, inflammation, and dysregulation of iron homeostasis simultaneously and could be important in the treatment of COVID-19 [102,103,104,105].

In fact, several studies have been conducted to evaluate the antiviral effect of Lactoferrin. It has been shown that asymptomatic, pauci-symptomatic, and moderate COVID-19 lactoferrin-treated patients show faster virus negativization and more rapid clinical recovery than untreated patients. Furthermore, Lactoferrin treatment is safe and well tolerated in all treated patients [106,107].

#### 3.2.6. Hesperidin

Hesperidin is a flavonic glycoside with antioxidant and anti-inflammatory [108] properties. It is commonly found in lemon and sweet oranges and acts against the influenza virusb, by inhibiting viral replication [109,110]. Combined with Quercetin, it has been recently proposed as a treatment to block the replication of SARS-CoV-2 by interfering with its interaction with the angiotensin 2 receptor converting enzyme [111,112,113,114,115]. Furthermore, it has been shown that Quercetin and vitamin C act synergistically against SARS-CoV-2 [116]. In a very recent paper, an early therapy with Hesperidin and quercetin, administered together with a non-steroidal anti-inflammatory drug with antiviral properties (indomethacin) and with an anti-aggregating drug (low-dose aspirin), was proposed to be carried out within 3 days from the beginning of the symptoms of SARS-CoV-2. This therapy showed a reduction in the severity of COVID-19 and rate of hospitalization [117].

In conclusion, regardless of the therapeutic activity of each micro or macronutrient, the nutritional status of COVID-19 patients should be carefully evaluated using standardized methods. In fact, all COVID-19 patients, especially those hospitalized or admitted to intensive care, should be considered at risk of malnutrition. Through a careful analysis of the real deficiencies and severity of COVID-19 disease, a correct integration should be administered without incurring the risk of refeeding syndrome [118].

The importance of performing a close community nutritional surveillance is highlighted by the nutritional support recommendations for COVID-19 patients provided by a professional clinical nutrition [119].

### 3.3. Nutrients Post COVID-19

At the end of acute phase of SARS-CoV-2 disease, so-called “long COVID” can develop, characterized by a series of persistent symptoms that last more than 12 weeks from the beginning of the infection [120].

Cognitive dysfunction and fatigue are main symptoms accompanied by sleep disturbances, lack of concentration, depression, and pain. Changes in taste and smell, headache, dizziness, coordination difficulties, memory loss, anxiety, and insomnia are also found. [121,122].

A clear explanation for these neurological symptoms has not yet been proposed. One possibility is that the virus can cross or damage the blood–brain barrier (BBB). In addition, the virus could enter the nose, and, via the olfactory nerve, arrive at the brain [123].

Fatigue appears to be independent of the severity of symptoms that characterize the acute phase of the disease [124]. In fact, long COVID also affects many healthy young people who have not been hospitalized [125,126,127].

It has been shown that the nutritional status of patients is important in determining the outcome of many diseases. This is the also case of COVID-19 [126,127].

COVID-19 patients, particularly those hospitalized and admitted to intensive care, who have developed metabolic disorders, have a poor nutritional status [128] due to malnutrition and weight loss. Simultaneously they suffer dyspnoea, nausea, vomiting, anorexia, dysphagia, diarrhea, and frailty as well as, sometimes, other comorbidities and prolonged hospitalization in intensive care [129,130,131].

To date, no guidelines for post-COVID patients have been yet provided. However, as it is known that dietary imbalances can adversely affect cognitive functions, causing worsening in reasoning, attention, and memory skills and promoting dementia and depression, it would be necessary to define them as soon as possible [132,133,134,135].

Nutrients, including vitamins B1, B6, B9, B12, C, D, and E, ω-3 fatty acids, and minerals, such as iron, zinc, and selenium, are known to play an important role in protecting against neuroinflammation and oxidative stress. Therefore, they have a very positive effect on cognitive functions [136,137,138,139,140,141,142,143,144,145,146,147].

Frequently, long COVID patients report the typical sensation of “brain fog”. The pathogenesis of brain fog is not yet fully understood. It could be induced by neuroinflammation caused by infectious agents, including SARS-CoV-2, stimulating mast cells to release microglia-activating mediators that in turn inflame the hypothalamus [148,149,150,151]. Therefore, inhibition of mast cells could be useful in treatment of brain fog. Natural flavonoids, including luteolin and quercetin, could be used as mast cell inhibitors: they inhibit neuroinflammation and decrease cognitive decline. In particular, luteolin is able to better penetrate the brain and inhibits both microglia and mast cells [152,153,154,155] (Figure 2).

## 4. Discussion

As mentioned above, the development of new variants of SARS-CoV-2 had a negative impact on the efficacy of vaccines and monoclonal antibodies therapy.

Thus, due to the extensive spreading of SARS-CoV-2 and the severity of the disease in some patients, it is essential to identify molecules capable of fighting against SARS-CoV-2 infection and treating COVID-19 disease. In particular, it is also worth noting that patients with mild symptoms may develop severe consequences, due to the onset of a series of long-lasting symptoms, constituting the so called long COVID.

In this review we have discussed a number of studies showing that nutrition may play an important role in influencing both the susceptibility and the clinical course of COVID-19 and long COVID, as is already known for other viral diseases.

We have shown that nutritional status plays a pivotal role in the function of the immune system, supporting both innate and adaptive immunity, influencing the proliferation and activity of immune cells. Furthermore, we highlighted that nutrients play a role in reducing inflammation. They inhibit leukocyte chemotaxis, inflammatory cytokine production, and T lymphocyte reactivity [24,25,26,35,37,38]. Moreover, they give rise to resolvins and protectins, which participate in the resolution of inflammation by normalizing excessive immune reactions [39]. In addition, they are essential for keeping intact and functioning tissue barriers. Finally, some of them seem to influence viral replication and they have even been shown to have a neuroprotective effect in long COVID, by decreasing cognitive decline.

Considering that the literature is often contradictory and that the reference to isolated studies could lead to false conclusions on COVID-19 prevention and treatment, at the moment, we can only suggest the introduction of micro and/or macronutrients into a balanced diet.

Indeed, up to now, no solid evidence supports the adoption of specific nutritional therapies, so it is necessary to await the results of the ongoing clinical trials.

Of course, since all forms of malnutrition negatively affect the functioning of the immune system, malnutrition will also impact the susceptibility to COVID-19. Of note, it has been shown that patients with obesity, heart disease, hypertension, or diabetes, if affected by COVID-19, have more severe infections with higher rates of hospitalization and mortality [156,157]

On the other hand, it should be considered that micronutrient amount is not routinely measured upon admission to hospital. Thus, public health strategies aimed at preventing micronutrient deficiencies, malnutrition, and over-nutrition remain of basic importance.

It should also be considered that, up to now, data on the recommended daily allowance (RDA) and adequate intake (AI) of dietary supplements to prevent or treat COVID-19 are not sufficient. They are usually customized for women, men, age groups, and for specific conditions. For instance, the effects of dietary supplement may be dependent on gender. Louca et al. observed a significant correlation between diet supplement intake and SARS-CoV-2 positivity in women, but no clear benefit was shown in men [158,159]. Moreover, many nutrients in very high doses can be dangerous.

Unfortunately, there are not conclusive data in this field, but many studies are ongoing. Mainly, studies on the use of micro and macronutrients in COVID-19 patients should be improved. If effective, nutrients could have the potential to change the course of the COVID-19 pandemic.

Last but not least, the use of micro and macronutrients is a relatively inexpensive and easy to manage treatment, needing no hospitalization.

In conclusion, large randomized controlled studies are needed to establish the real role of micro- and/or macronutrients in the different phases of COVID-19 and to test their positive and/or adverse effects, before the approval of their therapeutic use in this pathology.

## Data Availability

Not applicable.

## Abbreviations

COVID-19	Coronavirus Disease-2019
SARS-CoV-2	severe acute respiratory syndrome Coronavirus 2
WHO	World Health Organization
ACE2	angiotensin converting enzyme 2 receptor
S protein	Spike protein
TMPRSS2	transmembrane protease serine 2
IFN-β	interferon beta
CXCL-1O	CXC motif chemokine ligand 10
ARDS	acute respiratory distress syndrome
Se	selenium
Fe	iron
Zn	zinc
N-3 PUFAs	omega-3 fatty acids
EPA	enzymatic conversion of omega-3 eicosapentaenoic acid
DHA	docosahexaenoic acid
SPM	pro-resolution mediators
Lf	Lactoferrin
